# Quick-FLIC: validation of a short questionnaire for assessing quality of life of cancer patients

**DOI:** 10.1038/sj.bjc.6601782

**Published:** 2004-04-06

**Authors:** Y-B Cheung, C Goh, L-C Wong, G-Y Ng, W-T Lim, S-S Leong, E-H Tan, K-S Khoo

**Affiliations:** 1 Division of Clinical Trials and Epidemiological Sciences, National Cancer Centre, 11 Hospital Drive, Singapore 169610; 2 Department of Palliative Medicine, National Cancer Centre, 11 Hospital Drive, Singapore 169610; 3 Department of Therapeutic Radiology, National Cancer Centre, 11 Hospital Drive, Singapore 169610; 4 Department of Medical Oncology, National Cancer Centre, 11 Hospital Drive, Singapore 169610

**Keywords:** quality of life, shortening

## Abstract

A practically useful measure of quality of life should be simple and quick to complete. A shortened Chinese version of the Functional Living Index – Cancer (FLIC) was recently proposed and was called Quick-FLIC. This study aims to assess the measurement properties of the Quick-FLIC. A total of 190 patients who received care from the National Cancer Centre of Singapore completed a questionnaire package at baseline. Patients filled in a retest questionnaire on average 2 weeks after baseline to assess test–retest reliability and responsiveness to change. The Quick-FLIC scores correlated well with the Functional Assessment of Chronic Therapy – General scores (*r*=0.78). Patients with different treatment status, performance status and self-rated health had significantly different Quick-FLIC scores in the expected directions (ANOVA; each *P*<0.001). Internal consistency (Cronbach's alpha=0.87) and 2-week test–retest reliability (intraclass correlation=0.81) were also satisfactory. The measure was responsive to changes in health status (*P*<0.001). The Quick-FLIC is a valid and reliable measure of health-related quality of life of cancer patients. The shortening of established health-related quality of life instruments should be considered in order to reduce the burden of having patients to answer lengthy questionnaires.

For a health-related quality of life (HRQoL) measure to be clinically useful, it should be simple and quick to complete (Higginson and Carr, 2001). However, exisiting HRQoL questionnaires often include tens of items, which impose a burden on the part of patients and risk undermining response rate and data quality. In reviewing the problems in the assessment of HRQoL of cancer patients, Ballatori (2001) suggested that simpler HRQoL questionnaires that can be filled out in a shorter period of time are needed.

The concept of shortening a measurement scale with multiple (correlated) items by excluding less informative items is not new. It has been practised in the educational, psychological and, to a lesser extend, health research field (Coste *et al*, 1997). Ware *et al* (1996) successfully streamlined a generic quality of life measure – the Short Form 36 Health Survey – from 36 items to 12 items. Anne Moran *et al* (2001) developed a short version of the Chronic Respiratory Questionnaire without compromising the Questionnaire's measurement properties appreciably. A 12-item version of the General Health Questionnaire was developed from its longer parent versions, and was valued as a more usable measure in the older and frail populations (Cheung, 2002).

Although cancer researchers have repeatedly called for shorter HRQoL measurement scales (Bernhard *et al*, 1998; Ballatori, 2001; Gunnars *et al*, 2001), the shortening of HRQoL measure has received limited attention in the oncology field. A recent article proposed a shortened version of the Chinese version of the Functional Living Index – Cancer (FLIC) (Schipper *et al*, 1984; Goh *et al*, 1996), which is called Quick-FLIC (Cheung *et al*, 2003). Briefly, the cross-sectional study employed factor analysis to extract the most informative items from the Chinese version of the FLIC. A total of 11 items were selected to form the short measure: three related to physical well-being (‘feel well’, ‘pain or discomfort’ and ‘feel uncomfortable’), two related to each of the psychological (‘discouraged’ and ‘frightened of the future’), symptoms (‘nausea’ and ‘pain related to illness’), family (‘hardship on those closest to you’ and ‘satisfaction with work/housework’) and social aspects (‘willing to see friends’, and ‘willing to see those closest to you’). The score of each item ranged between 1 to 7. The sum of item scores was scaled to the 0–100 range to give a Quick-FLIC score (Fairclough, 2002, p.36)). The Quick-FLIC was compared with the original FLIC and the Functional Assessment of Cancer Therapy – General (FACT-G) (Cella *et al*, 1993) in the cross-sectional survey. It was found that the three measures have similar psychometric properties and they behaved similarly in relation to clinical variables. However, the study had two major limitations. Firstly, the Quick-FLIC was not employed as an individual measure. Instead, the original, 22-item FLIC was used and the subset of 11 items was identified from it. So the validity of the Quick-FLIC could have been elevated by a context effect. Secondly, the measure has not been tested in a longitudinal setting. The previous evaluation of this short measure was therefore incomplete. In this study we examine the validity, reliability and responsiveness to change of this new instrument, and discuss the value of the shortening of HRQoL instruments for oncology studies.

## MATERIALS AND METHODS

### Patients

From September 2002 to January 2003, patients who attended the outpatient clinic of the National Cancer Centre Singapore, as well as in-patients under the care of the Centre's department of palliative care, were recruited into the study. Patients were screened by the oncologists with the following inclusion criteria: able to understand written Chinese and speak Mandarin (the official Chinese language in Singapore), aged 18 years or older and physically fit to self-administer a questionnaire. Patients with evidence of brain metastasis, psychosis or severe depression were excluded. The study was approved by the Ethics Committee of the National Cancer Centre, Singapore.

### Design and procedure

Patients who passed the inclusion/exclusion criteria aforementioned were referred to a research coordinator who explained the study in detail, obtained informed consent, and delivered the baseline questionnaires. Patients self-administered and returned the questionnaires on the spot. The questionnaire package included the Quick-FLIC and the Chinese versions of FACT-G, as well as questions on marital status, education level and self-rated health. Self-rated health, which was known to be a powerful indictor of physical health and mortality (Cox *et al*, 1993), was measured on a five-point scale ranging from ‘very good’ to ‘very poor’. The median time to complete the baseline questionnaire package was 14 min. Karnofsky performance score, treatment status and some other clinical information were rated by the referring oncologists. At 1 week after the baseline, questionnaires, which consisted of the Quick-FLIC and some other health-related questions, were posted to the patients, together with a postage-paid envelope for returning it. Change in health status was measured on a five-point scale ranging from ‘much better’ to ‘much worse’. Patients were also asked whether they received chemotherapy, radiotherapy or pain medication since the baseline interview. The questionnaires included a question on the actual date the patients filled in the questionnaires. If the patients did not return the questionnaire after 2 weeks of the mailing, the research coordinator would gave them a telephone reminder. In addition, for patients who had a Karnofsky performance status below 80 at baseline and for in-patients, the research coordinator attempted to deliver the second questionnaire to them personally if they visited or remained at the location of care in the period from 1 to 2 weeks after the baseline. This extra effort was made because we expected that these patients might have a lower probability of responding to a questionnaire survey (Fairclough, 2002, pp.76–77).

The sample size planning considered several aspects of measurement properties. It turned out that the issue of responsiveness to change required the largest number of participants. To detect a change of 0.25 standard deviation in the pre- and post-test difference in Quick-FLIC scores in relation to a difference of one point on a five-point scale of change in health status, a sample size of 130 is required to achieve a power of 0.80 and an alpha of 0.05 (nQuery Advisor software, Elashoff, 2000). Assuming that 70% of patients who participated in the baseline study will return the retest questionnaire, a total sample size of 190 was required.

### Analysis

Missing values in the Quick-FLIC and FACT-G were imputed by the half-rule (Cella, 1997; Fairclough, 2002, p. 37). The characteristics of patients who provided complete and incomplete data were compared using Fisher's exact test since the latter group involved only a small number of people. Validity was examined by correlation analysis between Quick-FLIC and FACT-G, and by analysis of variance (ANOVA) of Quick-FLIC scores by categories of health and treatment variables. The ANOVA compared differences in mean Quick-FLIC values between groups of participants with different clinical characteristics, such as patients with or without present evidence of disease. A significant difference in mean values (*P*<0.05) was considered evidence for known-group validity. Although the ANOVA technique involves assumptions like normal distribution, it is robust to the violation of assumptions, especially when sample size is large (Rice, 1995). Education is often found to be a powerful predictor of general health and psychological well-being (Cox *et al*, 1993; Cheung, 2002). If Quick-FLIC specifically measures the impact of cancer and cancer treatment, it should not be strongly associated with this variable. Analysis of variance was used for this educational comparison. Reliability in terms of Cronbach's alpha was estimated in both the baseline and retest. Test–retest reliability was estimated by intraclass correlation. One estimate was limited to participants who filled in the retest questionnaire within 2 weeks from baseline, and one was limited to participants who rated themselves as having no change in health. There is no consensus on how to measure responsiveness to change and whether the change score, that is retest score minus baseline score, is a useful index. In particular, it has been suggested that the ‘residual gain score’, that is the actual retest score minus the retest score predicted by baseline score, is a better indicator of change (Senn, 1997; Streiner and Norman, 1989, pp. 133–134). To give a more comprehensive view, we investigated both residual gain scores and change scores in relation to change in health status by ANOVA and least-square test for trend. We compared those who received active treatment following baseline assessment with those who did not using ANOVA. Patients with a decline in health and patients who received active treatment since baseline were expected to have a larger decline in Quick-FLIC scores.

## RESULTS

### Patient characteristics

A total of 220 eligible patients were approached. In all, 17 of them refused to participate, 10 filled in about half of the questionnaires and then withdrew (four saying that it was too tiring); three left more missing values than computable by the half-rule. As such, there were 190 successfully completed questionnaires and 13 incomplete questionnaires.

Table 1

**Table 1:** Patient characteristics

		**Completed questionnaire (*n*=190)**		**Incomplete questionnaire (*n*=13)**
**Variable**	**Category**	**Frequency**	**Percentage**	**Frequency**
Gender	Female	99	52.1	7
	Male	91	47.9	6
				
Age (mean)		55		63
				
Education	Primary incomplete	52	27.4	8
	Primary complete	64	33.7	2
	Secondary	59	31.1	3
	Postsecondary	15	7.9	0
				
Tumour type	Breast	62	32.6	4
	Colorectal	35	18.4	2
	Lung	29	15.3	2
	Head and neck	35	18.4	3
	Others	29	15.2	2
				
Karnofsky score	100	49	25.8	1
	90	87	45.8	2
	80	24	12.6	5
	60–70	19	10.0	2
	30–50	11	5.8	3
				
Treatment status	Active treatment	87	45.8	10
	Surveillance	103	54.2	3
				
Presence of disease	Yes	81	42.9	12
	No	108	57.1	1
				
Type of care	Outpatient	178	93.7	6
	In-patient	12	6.3	7
				
Self-rated health	Very good	32	16.8	0
	Good	56	28.5	2
	Average	77	40.5	5
	Poor	14	7.4	3
	Very poor	11	5.8	2

gives a summary of patients’ characteristics at baseline. About half of the 190 patients were female; the mean age was 55 years. In total, 54% of the participants were under surveillance; 57% had no present evidence of disease. The distribution of performance status more concentrated on 90 (46%) and 100 (26%), but it spread over a wide range. As the number of participants with Karnofsky score below 60 was small (*n*=11, 6%), they were grouped into a single category. Most of the participants were outpatients. About 40% rated their health as average. Profile of the 13 patients with incomplete information was also given in Table 1. Percentages are not given as they are unstable when the number of observation is small. Those who did not fully complete the questionnaires were more likely to have lower performance status (*P*=0.003), on active treatment (*P*=0.043), and be in-patients (*P*=0.001).

### Descriptive statistics

Table 2

**Table 2:** Descriptive summary of quick-FLIC

	**N**	**Mean**	**s.d.**	**% at ceiling**
Quick-FLIC score	190	76.3	18.4	5
*Individual items*^a^				
1. Feel well	190	5.2	1.4	22
2. Hardship to those closed to you	190	5.6	1.9	51
3. Discourage	190	5.7	1.6	46
4. Satisfaction with work/housework	188	5.2	1.7	31
5. Feel uncomfortable	190	5.8	1.6	54
6. Pain or discomfort affects activities	190	5.3	1.9	39
7. Willing to see those closest to you	189	6.2	1.3	63
8. How much nausea	190	6.5	1.2	79
9. Frightened of the future	190	5.8	1.7	52
10. Willing to see friends	187	5.7	1.6	49
11. Pain or discomfort	189	4.4	2.4	35

a All items recoded to the direction of a higher score indicates a better quality of life. Scores on individual items ranged from 1 to 7; combined Quick-FLIC score scaled to range from 0 to 100.

provides the descriptive information of the Quick-FLIC and its individual items, which were recoded so that a high score indicates a better quality of life. The mean (s.d.) of Quick-FLIC scores was 76.3 (18.4). A large range of the Quick-FLIC scale was actually utilised, the minimum and maximum being 13.6 and 100, respectively. The individual items were more skewed, with up to 79% of participants reaching the ceiling value of one item (how much nausea?).

### Validity

**Figure 1:**
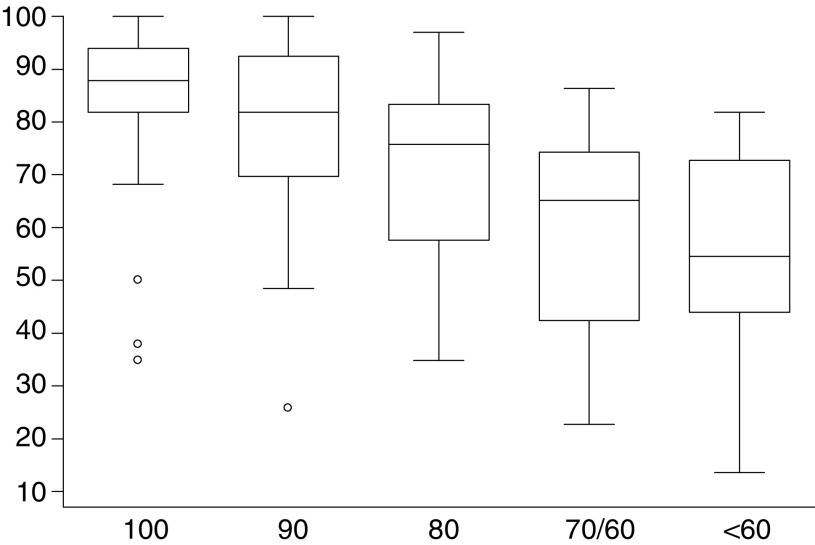
Box-whisker plot of Quick-FLIC scores by Karnofsky performance status.

The Quick-FLIC was highly correlated with FACT-G, with Pearson's correlation coefficient (*r*) being 0.78. (95% CI=0.71–0.83). Figures 1

and 2

**Figure 2:**
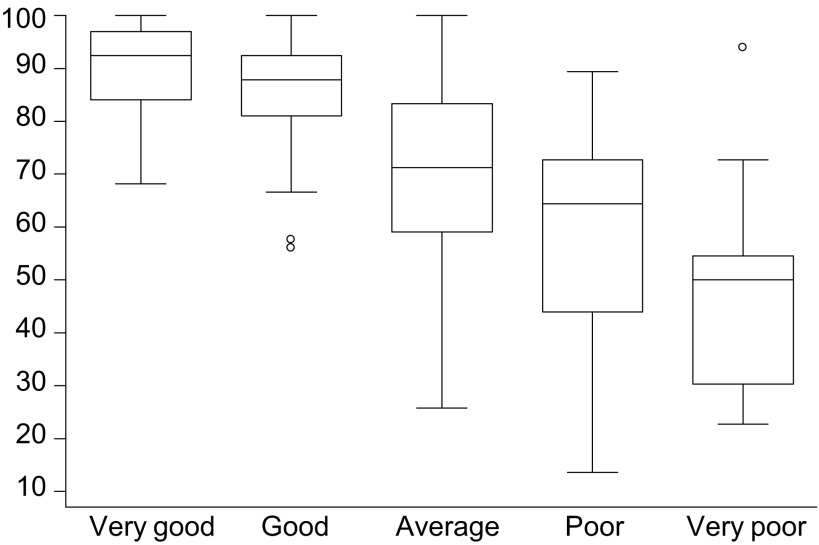
Box-whisker plot of Quick-FLIC scores by self-rated health status.

plot the Quick-FLIC scores in relation to Karnofsky performance score and self-rated health status. Both show a significant decrease in HRQoL as performance or health status declined (ANOVA; each *P*<0.001). In addition, as shown in Table 3

**Table 3:** Mean (s.d.) of quick-FLIC scores by patient characteristics

	**Present evidence of disease**	**Receiving active treatment**	**Secondary education or higher**
No	83.2 (14.7)	84.1 (14.1)	76.5 (18.3)
Yes	67.4 (19.2)	67.2 (18.8)	76.1 (18.8)
*P*-value (ANOVA)	<0.001	<0.001	0.889

, patients with present evidence of disease and patients receiving active treatment had significantly lower scores (each *P*<0.001). There was no significant difference in Quick-FLIC scores between educational groups (*P*>0.05).

### Responsiveness to change

Table 4

**Table 4:** Mean (s.d.) residual gain score and change score in relation to change in health and postbaseline treatment status

			**Residual gain score**		**Change score**	
**Variables**	**Category**		**Mean**	**s.d.**	**Mean**	**s.d.**
Change in health status	Much better	(*n*=44)	5.1	9.9	−0.2	10.6
	Better	(*n*=44)	2.9	10.0	−0.3	10.5
	Same	(*n*=56)	−2.7	12.0	−5.2	12.5
	Worse	(*n*=12)	−10.2	15.8	−10.5	18.7
	Much worse	(*n*=4)	−18.5	20.6	−20.8	23.6
	*P*-value (ANOVA)		<0.001		0.001	
						
Received active treatment	No	(*n*=100)	2.6	10.0	−1.7	10.4
after baseline	Yes	(*n*=59)	−4.3	15.2	−5.8	16.2
	*P*-value (ANOVA)		0.001		0.052	

Residual gain score=actual retest score minus retest score predicted by baseline score; change score=actual retest score minus baseline score.

shows the residual gain scores by changes in health status since baseline interview. Those who reported better or much better health had a Quick-FLIC score in the retest 2.9 and 5.1 points higher than predicted by their baseline scores; those whose health became worse or much worse had scores 10.2 and 18.5 points lower than predicted. The difference in residual gain score was more obvious in the lower range of change in health status than in the upper range, but a test for nonlinearity failed to show significance (*P*>0.05). A test for linear trend shows that for each step of decline in health status, the Quick-FLIC residual gain score decreased by 6.3 points (*P*<0.001). Furthermore, patients who did not receive active treatment since baseline had a mean residual gain score of 2.6 points higher than expected, while those who did had a mean of −4.3 points (*P*=0.001). Analysis of change score showed a similar pattern of responsiveness. A test for linear trend shows that for each step of decline in health status, the change score decreased by 3.7 points (*P*<0.001). The difference in change scores between groups with different postbaseline treatment status was marginally significant (*P*=0.052).

### Reliability

In all, 84% of the participants returned the retest questionnaires (*n*=160). Cronbach's alpha was 0.87 (95% CI=0.84–0.90) in the baseline and 0.88 (0.85–0.90) in the retest. The median interval from baseline to filling in the retest questionnaire was 14 days. The first and third quartiles were 10 and 21 days, respectively. Among participants who completed the retest within 14 days (*n*=91), the test–retest reliability was 0.81 (95% CI=0.73–0.88). Among patients who reported ‘the same’ health status (*n*=56) in the retest, the reliability was 0.78 (0.67–0.88). We also explored the test–retest reliability in patients who reported ‘much better’, ‘better’, ‘worse’ and ‘much worse’ health status. The coefficients were, respectively, 0.54, 0.77, 0.51 and 0.00 (The last one was truncated at zero since the error variance was larger than the between-patients variance [Fayers and Machin, 2000]).

## DISCUSSION

Despite substantial development in quality of life research in the last two decades, practical problems remain to be solved. It has been suggested that the use of shorter instead of longer HRQoL questionnaires might mitigate the problems of patients’ inability/unwillingness to complete a questionnaire because of poor health and reduce missing values during follow-up (Bernhard *et al*, 1998; Axelsson and Sjoden, 1999; Ballatori, 2001; Higginson and Carr, 2001). In our study, it was also patients who were frailer who tended to provide incomplete questionnaire information. Some previous studies have demonstrated the practicability of reducing the number of items in composite measurement scales by 60–70% (Ware *et al*, 1996; Anne Moran *et al*, 2001). Although relatively common in other areas of research and services, the shortening of existing HRQoL measures in oncology has not received much attention.

A recent cross-sectional study proposed a short version of FLIC. The present study has formally evaluated its properties and found it satisfactory in various aspects. Nevertheless, the present study was not designed to show the practical advantages of using a short or shortened HRQoL measure *vs* a long or original one. So, for instance, we cannot answer whether using a shorter instrument would indeed give fewer missing values. Another limitation was that the number of patients who reported a decline in health status in the retest was relatively small, and the change in health status variable was based on self-assessment. Further studies with a larger number of respondents in the lower range of changes in health and with objectively assessed changes in health status would be helpful. Some individual items were quite skewed; one of them having 79% of participants at the ceiling value. This is a quite common phenomenon in quality of life assessment. Streiner and Norman (1989, p. 44) recommended that items with 80% or more respondents endorsing one category should be excluded from a composite scale. The Quick-FLIC items did not exceed this level and their distributions did not appear to be more concentrated than other cancer HRQoL measures’ (eg Basen-Engquist *et al*, 2001). However, we do not recommend using a few skewed items to assess subdomains of HRQoL; we use all the 11 items to assess overall HRQoL. In order to improve response rate to the retest questionnaire, we made an effort to deliver the retest questionnaire to the patients personally rather than by post if they had poor performance status or if they were in-patients at baseline. Without doing a randomised comparison, we cannot tell how successful the extra effort was on response rate. However, the procedure should not affect the measurement properties shown here, as the retest data was not evaluated in relation to baseline clinical characteristics.

The correlation between Quick-FLIC and FACT-G (*r*=0.78) in this study was very similar to that between FLIC and FACT-G (*r*=0.79) in a large-scale study that used the FLIC to validate the FACT-G (Cella *et al*, 1993), giving support to the concurrent validity of Quick-FLIC. Furthermore, trends of Quick-FLIC score in relation to health status assessed by patients and performance status assessed by clinicians were clearly seen. Its known-group validity was supported by its ability to clearly differentiate patients with different treatment and disease status. That it did not relate to education suggested the Quick-FLIC measured HRQoL that was quite specific to cancer-related problems rather than general health or well-being. It was suggested that a measurement scale should have a test–retest reliability of 0.75 or above (Streiner and Norman, 1989, p. 90). Quick-FLIC passed this standard and its reliability in terms of Cronbach's alpha was high. Both residual gain score and change score showed its responsiveness to change in health status. Postbaseline treatment status was also reflected in the change in residual gain scores.

Change in health was positively related to baseline health status. For instance, a large proportion of participants in the ‘much better’ (77%) and ‘better’ (43%) groups had rated their health as either ‘good’ or ‘very good’ in the baseline interview and they had high baseline Quick-FLIC scores. As there was not much room to improve on the Quick-FLIC scale for these participants, the means of change scores were close to zero rather than showing a positive change. This is a ceiling effect that may suppress an indication of improvement among well patients. Furthermore, test–retest reliability was higher among participants who had ‘much better’ health status in the follow-up survey than those who had ‘much worse’ (0.54 *vs* 0.00). Similarly, it was higher among participants who had ‘better’ health status than those who had ‘worse’ (0.77 *vs* 0.51). This again indicated a ceiling effect. In light of it, analysis of residual gain score is more suitable than analysis of change score in the assessment of changes in HRQoL in patients who are quite well at baseline (Senn, 1997; Streiner and Norman, 1989, pp. 133–134). Cancer research often examines the changes in HRQoL of patients with symptoms and/or under therapy at baseline, rather than patients without symptoms or patients who are under surveillance only. So this ceiling effect should not have a big practical impact on the applications of the Quick-FLIC.

Ethnic Chinese people reside in many regions of the world. In Singapore and China, they use the simplified form of Chinese characters. The Quick-FLIC needs to be changed into the conventional form of characters before use in areas like Hong Kong and Taiwan. This is a simple task since most Chinese word-processing programmes allow users to choose the type of characters. However, due to differences in factors such as literacy level, further studies outside Singapore are required before the usefulness of the Quick-FLIC in these regions can be assured. One of the present authors translated the English FLIC into Chinese and Malay (Goh *et al*, 1996). The Chinese FLIC forms the basis of the present work. So there is no need to repeat the translation process to develop English and Malay versions of the Quick-FLIC. We can simply take the corresponding items from the English and Malay version of FLIC. However, the resultant short questionnaires should be subject to validation again in English- and Malay-speaking populations.

Even though only half of the 22 items of the original FLIC are included to form the Quick-FLIC, we have found it to have sufficient validity, reliability and sensitivity as a measure of overall health-related quality–of life measurement scale for use in oncology. In this case, the idea of shortening an existing well-established HRQoL measurement scale appears to be useful. The advantages of using short instruments may include: less burden on the part of patients, fewer missing values and/or incomplete questionnaires, higher willingness to participate. While the first advantage seems to be definitional and worthy in its own sake, evidence supporting the latter two claims is scanty and the present study does not provide such evidence either. In summary, we have found the Quick-FLIC a satisfactory measure of health-related quality –of life of cancer patients. We recommend further research on the practical advantages of using this and other short instruments in comparison with longer HRQoL instruments.
